# Comprehensive Transcriptome of the Maize Stalk Borer, *Busseola fusca*, from Multiple Tissue Types, Developmental Stages, and Parasitoid Wasp Exposures

**DOI:** 10.1093/gbe/evaa195

**Published:** 2020-09-18

**Authors:** The Busseola fusca Genome Consortium

**Keywords:** agricultural pest, Lepidoptera, insect genomics

## Abstract

*Busseola fusca* (Fuller) (Lepidoptera: Noctuidae), the maize stalk borer, is a widespread crop pest in sub-Saharan Africa that has been the focus of biological research and intensive management strategies. Here, we present a comprehensive annotated transcriptome of *B. fusca* (originally collected in the Western Province of Kenya) based on ten pooled libraries including a wide array of developmental stages, tissue types, and exposures to parasitoid wasps. Parasitoid wasps have been used as a form of biocontrol to try and reduce crop losses with variable success, in part due to differential infectivities and immune responses among wasps and hosts. We identified a number of loci of interest for pest management, including genes potentially involved in chemoreception, immunity, and response to insecticides. The comprehensive sampling design used expands our current understanding of the transcriptome of this species and deepens the list of potential target genes for future crop loss mitigation, in addition to highlighting candidate loci for differential expression and functional genetic analyses in this important pest species.

## Introduction

**Fig. 1.**
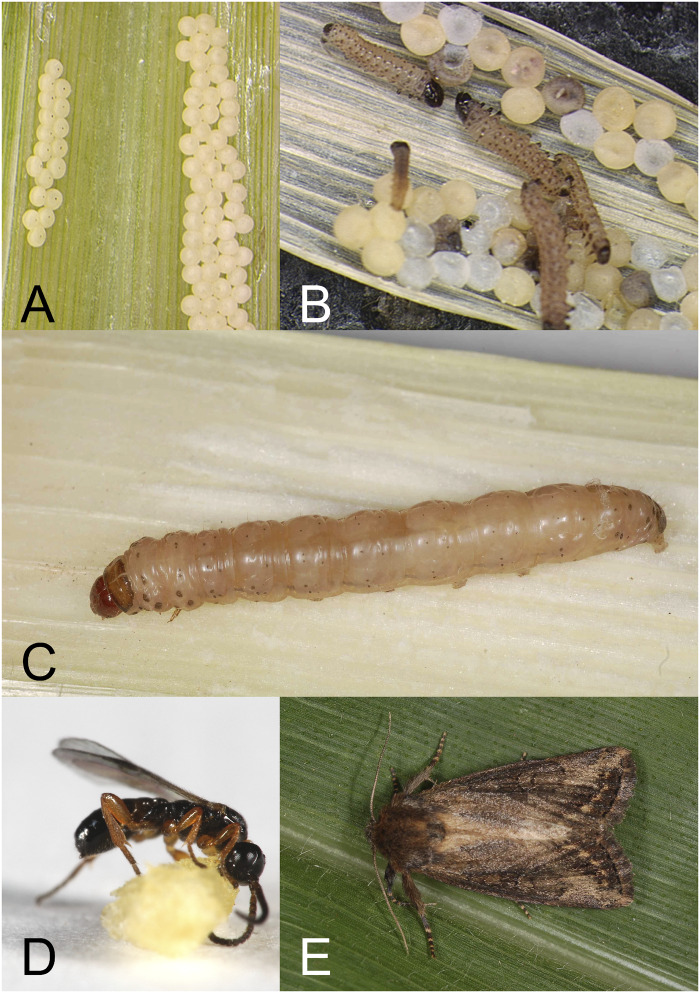
*Busseola fusca* (*A*) eggs, (*B*) neonates after hatching, (*C*) fourth instar larva, (*D*) parasitoid wasp, *Cotesia sesamiae* (female), used for exposure treatment, and (*E*) adult (female).

The maize stalk borer, *Busseola fusca* (Fuller) (Lepidoptera: Noctuidae; fig. 1), is an important pest of cereal crops in sub-Saharan Africa. Due to its abundance and distribution, it represents a major constraint to food production where infestation rates are high (Kfir et al. 2002). Crop losses resulting from *B. fusca* feeding activity vary by region, but can result in a total loss in some areas (Van den Berg et al. 1991, Calatayud et al. 2014). Its impact on the food security and economic well-being of people has led to a number of studies into the physiology and ecology of the species (reviewed in Calatayud et al. [2014]). With the release of the genome (from the same lineage used in this study; Hardwick et al. 2019) and the publication of the first transcriptome (derived from neonate tissue from a genotype collected in South Africa; Peterson et al. 2019), we now have basic genomic information for this species. With pest species, in particular, the identification of candidate genes related to important traits such as developmental timing, reproduction, insecticide resistance, adaptation to plant defense mechanisms, immunity, and chemoreception requires sampling multiple time points, exposures, and populations. This is particularly relevant in *B. fusca*, where certain strains are known to be differentially vulnerable to infection by parasitoid wasps (Kfir 1995). This information, in turn, can inform or enhance management strategies (e.g., those developed for *Plutella xylostella*; You et al. 2013).

Female *B. fusca* typically deposit eggs (fig. 1*A*) between the stem and leaf sheet of the host plant. Larvae hatch (fig. 1*B*), feed on young leaves, and penetrate the plant stem during the third and fourth instar (fig. 1*C*) where they remain until pupation. Feeding during the larval stage, which is also when the animals are vulnerable to parasitoid wasps (fig. 1*D*), damages the host plant, and reduces yield or kills the plant. After pupation, adult moths (fig. 1*E*) use chemosensory cues and receptors to attract and find mates, food, and suitable places to lay eggs. Pest management strategies can include, for example, introducing substances that can interrupt sending or receiving chemosensory cues to/from mature adults or can include the introduction of biocontrol species to which the focal species is unable to mount a defense response. Because it involves fewer chemicals, biological control is a pest management strategy that has been employed with *B. fusca* over the last several decades. Beginning in the 1990s, a biocontrol program was launched in Kenya using the indigenous larval parasitoid of *B. fusca*, *Cotesia sesamiae* (Cameron; Hymenoptera: Braconidae; fig. 1*D*). Prevalence of parasitism by *C. sesamiae* can vary (ranging from <5% to 75%; Kfir 1995; Sallam et al. 1999; Jiang et al. 2006; Songa et al. 2007), in part due to differences among wasp strains (Mochiah et al. 2002; Gitau et al. 2010; Branca et al. 2011). *Busseola fusca* are resistant to infection by *C. sesamiae* from Mombasa (coastal Kenya), referred to hereon as Cs-Coast, but vulnerable to *C. sesamiae* from Kitale (a site in inland Kenya; referred to hereon as Cs-Inland; Ngi-Song et al. 1995).

Here, we present a comprehensive transcriptome of *B. fusca* based on data from ten libraries representing different tissues, developmental stages, and parasitoid-exposure treatments. Phylogeographic studies (Sezonlin et al. 2006; Dupas et al. 2014) and reports of dominant inheritance of field-evolved resistance to *Bt* maize (Campagne et al. 2013) represent the current frontier, in terms of studies of individual genes. We use the transcriptome to identify important candidate genes and gene families, as well as investigating the transcriptomic profiles of nongenic regions, for future research and ongoing management efforts aimed at curbing the effects of this devastating crop pest.

## Materials and Methods

See supplementary methods, Supplementary Material online, for complete details. Briefly, sample collection, preparation, and sequencing were performed at the International Centre of Insect Physiology and Ecology (*icipe*) and Biosciences eastern and central Africa genomics facility at the International Livestock Research Institute in Nairobi, Kenya (BecA-ILRI Hub). Specimens were obtained from a colony initiated from larvae collected in maize fields from the Western Province of Kenya in 2008, and maintained on an artificial diet under laboratory conditions at *icipe*. RNA extractions were performed on ten different *B. fusca* tissues types, (thoraces, antennae [both sexes], and female ovipositor), developmental stages (eggs, neonates, fourth instar larvae, and adults [both sexes]), and parasitoid-exposure conditions (unexposed and exposed to two strains of wasp; supplementary table S1, Supplementary Material online). We used a TruSeq RNA Library Prep Kit (Illumina) to prepare libraries and performed next-generation sequencing using Illumina MiSeq technology, resulting in ten libraries of 300-bp paired-end reads (supplementary table S2, Supplementary Material online). Sequence data are available on NCBI SRA (BioProject: PRJNA553865). We used the RNA-seq de novo assembly program Trinity (version 2.4; Grabherr et al. 2011) to assemble the transcriptome (table 1), and generated a set of nonredundant unigenes from our transcripts using the program CD-HIT (version 4.7; Li and Godzik 2006; Fu et al. 2012). The transcriptome assembly is available on NCBI (BioProject: PRJNA553865). We also assembled each library individually, and generated library-specific sets of nonredundant unigenes with CD-HIT. We ran BUSCO (version 3; Waterhouse et al. 2018) to assess the completeness of our assembly and Trinotate (v. 3.0.2; Bryant et al. 2017) to annotate the transcriptome. We quantified the distribution of genes within different gene ontology (GO) categories using WEGO (version 2.0; Ye et al. 2018; supplementary fig. S1, Supplementary Material online). Finally, we used OrthoFinder (version 2.3.3; Emms and Kelly 2019) to identify orthologous gene clusters among Lepidopteran transcriptomes and identified the 20 largest gene families in *B. fusca.* We identified the top 25 most highly expressed transcripts for each RNA library by mapping reads from each library to the pooled transcriptome assembly unigenes using the program STAR (version 2.7; Dobin et al. 2013) and generated unique lists of the 25 transcripts with the highest RPKM values for each.

**Table 1:** Assembly Statistics for *Busseola fusca* Transcriptome (Bioproject PRJNA553865) from ten Pooled Libraries Derived from Tissue Sampled from Specimens Originally Collected in Western Province, Kenya

	Transcriptome (Full Assembly)	Transcriptome (Unigenes)
Total size (bp)	171,925,619	116,455,446
Number of transcripts	240,022	185,159
Number of genes	129,814	123,344
Number of protein-coding genes	23,374	22,707
Number of proteins	59,341	39,445
Maximum transcript length (bp)	28,079	28,079
Median transcript length (bp)	398	377
% GC	40.1	40.2
% BUSCO genes—Eukaryota	99.6	99.6
% BUSCO genes—Arthropoda	99.1	99.2
% BUSCO genes—Insecta	98.7	98.6

## Results

Our *B. fusca* transcriptome (table 1 and supplementary table S2, Supplementary Material online; BioProject PRJNA553865), which contained >99% of the conserved single-copy orthologs used by BUSCO to assess completeness, contains approximately 1/3 more transcripts than previously known for this species, greatly expanding the number of genes available for further investigation (Peterson et al. 2019). The median number of isoforms per gene was one for all individual libraries, with library 5_S5_L001 (infection treatment: exposed Cs-Inland) having the highest maximum number of isoforms for a single transcript (transcript TRINITY_DN8760_c0_g1, with 45 isoforms reported by Trinity). Furthermore, the antenna libraries (4_S4_L001 and 3_S3_L001) had the highest percentage of genes with greater than one isoform per gene (21.1% and 19.4%, respectively), whereas the egg library (2_S2_L001) had the lowest (6.6%). We annotated the assembled *B. fusca* transcriptome using Trinotate and identified 22,707 protein-coding genes and generated a set of 39,445 proteins overall. We identified more protein-coding genes than were predicted in the previously published *B. fusca* whole-genome assembly (19,417; Hardwick et al. 2019). Of the 22,707 predicted protein-coding genes found in this study, 19,401 aligned to the genome assembly. The additional 4,306 genes identified in this study may represent alternative transcripts or fusion transcripts that the transcriptome annotation software does not recognize as variants produced by a given locus or loci. Alternatively, it possible these sequences were unrecognized by the genome annotation software because the depth of sequencing coverage for the whole-genome sequencing project was insufficient to fully assemble the genome given its size (∼500 Mb) and repeat content (∼50%).

Supplementary figure S1, Supplementary Material online, gives an overview of the GO terms associated with all annotated transcripts in the *B. fusca* transcriptome assembly after 20,118 of genes were assigned to GO categories using Trinotate. Using protein sets from *Bombyx mori*, *Manduca sexta*, and *P. xylostella*, we searched for orthologs in *B. fusca* and characterized gene families*.* The top 20 largest gene families identified by OrthoFinder are listed in supplementary table S3, Supplementary Material online. We report the most abundant transcripts across all libraries (supplementary table S4, Supplementary Material online) and in each individual tissue type, developmental stage, and infection treatment (supplementary tables S5–S7, Supplementary Material online) and between male and female antennae (supplementary table S8, Supplementary Material online) and thoraces (supplementary table S9, Supplementary Material online). We also characterize the nongenic transcriptomic profiles by reporting the diversity of transposable elements (TEs) transcribed in each library (supplementary table S10, Supplementary Material online) and the evidence for transcription of putatively horizontally transferred regions of the genome based on high identity between sequences in *B. fusca* and *C. sesamiae* (both strains; supplementary table S11, Supplementary Material online).

## Discussion

Because food security is an essential component of public health worldwide, managing pests that target major crop species is crucial (World Bank 2008). Here, we sequenced, assembled, and annotated the transcriptome of *B. fusca* to try and identify candidate genes for pest management strategies and to understand more about the biology of this species. This study expands on previously published transcriptomic resources in several ways. In particular, we analyzed data from ten different RNA-Seq libraries spanning multiple life stages, tissue types, and sexes. The inclusion of these groups allowed us to identify additional transcripts relative to previous studies. Our full transcriptome assembly includes 240,022 sequences. In contrast, the only other *B. fusca* transcriptome published to date using the larval stage only identified 170,756 transcript sequences (Peterson et al. 2019). The current study also expands on the geographical range of *B. fusca* populations surveyed; whereas the previously published transcriptome is based on tissues from individuals that originated in South Africa, our work focuses on individuals originating from Western Province, Kenya. The inclusion of these additional populations, developmental stages, and tissues will aid in the development of targeted pest control based on life cycle and behavior in this destructive species.

Based on our transcriptome (see supplementary methods and results, Supplementary Material online, for complete details), we identified genes of interest putatively involved in chemoreception. A number of the transcripts with potential chemosensory function were abundant in the antennae relative to other tissue types. Specifically, ten out of 25 transcripts uniquely highly expressed in the antennae had significant homology to general odorant-binding proteins (supplementary table S7, Supplementary Material online). General odorant-binding proteins are distributed in the sensilla, and function to bind odorants and transport them to odorant receptors. General odorant-binding proteins have been shown to be involved in the detection of plant volatiles (Vogt et al. 2002; Nardi et al. 2003; Maida et al. 2005; Liu et al. 2015). We also observed differences between male and female libraries in genes expressed in the antennae, which may be of interest for determining sex-specific chemosensory genes. For example, although both males and females showed high expression of general odorant-binding proteins, we identified suites of uniquely abundant general odorant-binding proteins that were distinct for each sex (supplementary table S8, Supplementary Material online). In addition, the female antenna library exhibited elevated abundance of two transcripts with high similarity to pheromone-binding protein. Pheromone-binding proteins are typically associated with the detection of female sex pheromones by males, but some studies have identified high levels of expression of the genes that produce these proteins in female antennae of moths in the family Noctuidae (Callahan et al. 2000). The abundance of these transcripts in females may suggest a role of pheromone-binding protein genes in detecting odorants other than sex pheromone in *B. fusca*.

We also sought to better understand immune response in *B. fusca*, by identifying transcripts expressed in larvae infected by the parasitoid *C. sesamiae*. *Busseola fusca* exhibits differential susceptibility to infection by *C. sesamiae* from coastal and inland subpopulations. To shed light on the biological mechanisms underlying this difference, we assessed gene expression in larva exposed to Cs-Inland and Cs-Coast, with the goal of determining immune genes highly expressed in each treatment. Fourteen of the 25 most highly expressed transcripts in the Cs-Coast treatment had significant sequence similarity to arylphorin (supplementary table S5, Supplementary Material online). Arylphorin was also the largest *B. fusca* gene family identified via our analysis of orthologous gene clusters (supplementary table S4, Supplementary Material online). Arylphorin is a storage protein produced throughout larval instars in Lepidoptera (Riddiford 1995), and has also been proposed to play a role in humoral immune defense in response to bacterial infection (Freitak et al. 2007). In contrast to the Cs-Coast treatment, larvae in the Cs-Inland treatment showed high expression of gloverin and lebocin, both of which have antimicrobial function in Lepidoptera (reviewed in Yi et al. [2014]). These results indicate that arylphorin, gloverin, and lebocin may be good targets for future research to understand why Cs-Inland are able to successfully parasitize *B. fusca* larva, whereas Cs-Coast are not.

We identified several large gene families potentially of interest for understanding *B. fusca*’s response to insecticides. One of the largest *B. fusca* gene clusters we identified was the xanthine dehydrogenase family, encompassing five genes and nine isoforms. Xanthine dehydrogenase is known to be involved in the production of nitrogenous excretory compounds (Bergmann and Dikstein 1955; Cochran 1985), and has also been implicated in phytochemical detoxification (Slansky 1993). Previous research has found that duplication of the xanthine dehydrogenase gene is related to resistance to organophosphorus insecticide resistance in mosquitoes (Coleman and Hemingway 1997). We also identified a large *B. fusca* gene family corresponding to the transient receptor potential (TRP) channel pyrexia gene. This family encompassed five genes and six isoforms. TRP genes are related to environmental perception in insects (Venkatachalam and Montell 2007; Fowler and Montell 2013), and are common targets of insecticides (Kwon et al. 2010; Nesterov et al. 2015; Kandasamy 2017). Additional research to identify the specific functions of the large xanthine dehydrogenase and TRP pyrexia gene families in *B. fusca* may shed light on the potential for insecticide resistance in this species, and inform future control efforts. Future studies may also utilize our transcriptomic resources to better understand Cry toxin resistance by looking at tissue-specific expression of genes such as alkaline phosphatase, aminopeptidase N, cadherin Cry toxin receptor, ATP-binding cassette transporters, and mitogen-activated protein kinases (Peterson et al. 2017).

We identified a number of highly abundant transcripts in the antennae which were annotated as replicase polyproteins (supplementary table S7, Supplementary Material online). Upon further investigation, these sequences had significant BLAST hits to iflavirus sequences. Iflaviruses are single-stranded RNA viruses that to date have been shown to exclusively infect arthropods (van Oers 2010). RNA derived from iflaviruses occurs at high concentrations in the antennae of infected individuals (Dos Santos et al. 2019). To our knowledge, iflavirus infection has not previously been observed in *B. fusca* and the significance of infection should be investigated further. To characterize the nongenic components of the transcriptome, we used the previously curated repeat library (from Hardwick et al. [2019]) to search against the transcriptomes (supplementary table S10, Supplementary Material online). The majority of TEs in *B. fusca* are expressed in each of the individual transcriptomes, with the greatest diversity transcribed in male antennae. We also used low-coverage draft sequences generated from the two parasitoid wasp strains (*C. sesamiae* Kitale and Mombasa) to identify candidate sequences that may have been horizontally transferred to or from *B. fusca* (supplementary data file S6, Supplementary Material online) and are transcribed (14 of 17 identified candidates; supplementary table S11, Supplementary Material online). Interestingly, three of the candidate regions appeared to be of bracoviral origin, suggesting that, as with many other insects (Gasmi et al. 2015), viruses may play an instrumental role in both the genomic and transcriptomic profiles of *B. fusca*.

## Conclusion

By sequencing a comprehensive set of ten libraries representing different developmental stages, tissue types, and parasitoid exposures, our transcriptome contains the largest number of expressed transcripts available to date and has allowed us to identify a number of loci of interest for potential pest management. Namely, loci potentially involved in chemoreception, response to infection, and response to insecticides could serve as promising targets for future research. We hope insights from the *B. fusca* transcriptome will aid ongoing efforts to develop control measures that can be deployed as part of an integrated pest management strategy in order to reduce *B. fusca*’*s* impact on food security across the continent.

## Supplementary Material

Supplementary data are available at *Genome Biology and Evolution* online.

## Supplementary Material

evaa195_Supplementary_Data
