# Supplementary material for: Comprehensive Transcriptome of the Maize Stalk Borer, Busseola fusca, from Multiple Tissue Types, Developmental Stages, and Parasitoid Wasp Exposures
Source: Genome Biol Evol. 2020 Sep 18;12(12):2554–60. doi: 10.1093/gbe/evaa195 (PMC7802516; doi:10.1093/gbe/evaa195)
Supplement: evaa195_Supplementary_Data [file evaa195_supplementary_data.zip › 092520_FINALBfusca_Transcriptome_SuppMaterials.docx]

**List of Supplemental Materials**

**Supplemental Methods**

**Supplemental Results**

**Supplemental References**

**Supplemental Figure 1.** GO term categories for transcripts annotated using Trinotate and classified into top level categories using WEGO.

**Appendix and Supplemental Tables,** uploaded separately in a single workbook:

**Appendix A.**  Full author list with institutional affiliations.

**Table S1.** Sample information for *Busseola fusca* RNA sequencing libraries.

**Table S2.** Summary statistics for transcriptomes generated from individual tissue and developmental stage-specific libraries.

**Table S3.** Twenty largest gene families in *Busseola fusca* identified by orthogroup analysis.

**Table S4.** Most abundant transcripts from across all pooled libraries prepared using *B. fusca*.

**Table S5.** Most abundant (top 25) transcripts from libraries prepared using *Busseola fusca* exposed to *Cotesia sesamiae* Mombasa and Kitale.

**Table S6.** Most abundant (top 25) transcripts from libraries prepared from different developmental stages of *Busseola fusca.*

**Table S7.** Most abundant (top 25) transcripts from libraries prepared using different tissues from *Busseola fusca*.

**Table S8.** Most abundant (top 25) transcripts from libraries prepared using *Busseola fusca* female and male antennae.

**Table S9.** Most abundant (top 25) transcripts from libraries prepared using *Busseola fusca* female and male thoraces.

**Table S10.** Transposable elements (listed by type) for which there is evidence of transcription in either the individual or comprehensive transcriptomes.

**Table S11.** Candidate horizontal transfer sequences shared between *B. fusca* and *Cotesia sesamiae* (sequenced from two strains, Kitale and Mombasa) that are transcribed in the individual and comprehensive transcriptomes.

**Supplemental Data Files,** uploaded separately in a single zipped file:

**Data File S1.** Trinity-generated file with assembled transcripts for pooled transcriptome.

**Data File S2.** Trinotate-generated file with transcriptome annotations.

**Data File S3.** Transdecoder-generated file of protein sequences.

**Data File S4.** Low coverage draft whole genome sequence of *Cotesia sesamiae* (Kitale).

**Data File S5.** Low coverage draft whole genome sequence of *Cotesia sesamiae* (Mombasa).

**Data File S6.** FASTA file of all horizontal transfer candidates between *Busseola fusca* and *Cotesia sesamiae* (Kitale and Mombasa) based on >95% sequence identity and a minimum length of 80 bp.

**All other data associated with this project can be found on NCBI under Bioproject PRJNA553865.** Sequence data and assemblies for individual libraries can be found under the following accessions: 2_S2_L001: SRR12107984, GITF00000000; 1_S1_L001: SRR12107983, GITJ00000000; S2_S1_L001: SRR12107982, GITA00000000; 5_S5_L001: SRR12107981, GITI00000000; S3_S2_L001: SRR12107980, GITB00000000; S10_S5_L001: SRR12107985, GISZ00000000; 4_S4_L001: SRR12107986, GITH00000000; 3_S3_L001: SRR12107987, GITG00000000; S7_S4_L001: SRR12107989, GITE00000000; S6_S3_L001: SRR12107988, GITD00000000. The comprehensive assembly is under accession GISL00000000.

**Supplemental Methods**

All animal rearing, tissue collection, library preparation, and sequencing were performed at the International Centre of Insect Physiology and Ecology (*icipe*) and Biosciences eastern and central Africa Hub genomics facility at the International Livestock Research Institute (ILRI) in Nairobi, Kenya (BecA-ILRI Hub). Briefly, specimens were obtained from a colony of *B. fusca* initiated from larvae collected in maize fields from the Western Province of Kenya in 2008, and maintained on an artificial diet of a mixture of brewer’s yeast, vitamins, sucrose, maize leaf powder and bean seeds (*Phaseolus vulgaris)* powder, suspended in agar (Onyango and Ochieng'-Odero 1994) under laboratory conditions (26 ± 1°C and 50-60% relative humidity) at the International Centre of Insect Physiology and Ecology (*icipe*) in Nairobi, Kenya. To rejuvenate the colony, wild individuals were collected from maize fields in Western Kenya and added three times a year. Pupae were sexed, and males and females kept separately in plastic boxes (21 cm long x 15 cm wide x 8 cm high) until adult emergence.

*RNA extraction and library preparation*

RNA extractions were performed on 10 different *B. fusca* tissues types, (thoraces, antennae [both sexes], and female ovipositor), developmental stages (eggs, neonates, fourth instar larvae, and adults [both sexes]), and parasitoid-exposure conditions (unexposed and exposed to two strains of wasp; Table S1). Briefly, for the parasitoid conditions, we exposed *B. fusca* larvae to female parasitoid wasps of *C. sesamiae* strains obtained from laboratory-reared wasp colonies of both Cs-Inland and Cs-Coast. The Cs-Inland strain was obtained from *B. fusca* larvae collected from maize fields in Kitale, Western Kenya, in 2006. The Cs-Coast strain was obtained from *Sesamia calamistis* larvae collected from maize fields in Mombasa (on the Kenyan coast) in 2007. Fourth instar larvae of *B. fusca* that were parasitized by Cs-Inland or Cs-Coast (as well as unparasitized larvae) were collected 6 h post-exposure to the parasitoids.

All samples were put in RNAlater stabilizing solution (Invitrogen cat. No. AM 7020) and kept at -80°C until RNA extraction. During RNA extraction, each sample was quickly ground into a fine powder with liquid nitrogen, and total RNA was prepared using TRIzol® Reagent (Invitrogen cat No 15596-026) with slight modifications. RNA quantification was performed by bioanalyzer RNA kits (Agilent Technologies) and quality was further confirmed by gel electrophoresis. We used a TruSeq RNA Library Prep Kit (Illumina) to prepare libraries for each sample, with an enrichment step to target mRNA. We then performed next-generation sequencing using Illumina MiSeq technology, resulting in ten libraries of 300 bp paired-end reads (Table S2). Sequence data are available on NCBI SRA (raw data will be added to Bioproject: PRJNA553865).

*Sequencing the transcriptome*

We sequenced all ten libraries using the Illumina MiSeq platform at the BecA-ILRI Hub in Nairobi, Kenya. To ensure that our RNA-Seq data set was high quality, we followed a modified version of the Oyster River Protocol for pre-processing RNA-Seq reads (MacManes 2018). First, we screened for PhiX contamination by removing reads that mapped to the PhiX genome. Next, we identified and corrected erroneous bases within our RNA-Seq data using the *k*-mer based method implemented in the program Rcorrector (Song and Florea 2015). Finally, we trimmed low quality and adapter sequences from our reads using the program Skewer (Jiang et al. 2014) with a 3’ end quality trimming threshold of 2, a mean quality cutoff of 2, and a minimum read length cutoff of 25bp. Pooling across the ten libraries, 108,076,310 reads remained after cleaning (Table S2). Our sequencing efforts produced 108,076,310 reads (300 bp long paired-end reads, in both cases; Table S2).

*Transcriptome assembly*

We used the RNA-Seq *de novo* assembly program Trinity (version 2.4; Grabherr et al. 2011) to assemble the pooled, cleaned data set into transcripts using the “--no_normalize_reads” parameter, which gave a more complete transcriptome (as assessed by metrics described below) when compared to an assembly using default parameters. Where applicable, Trinity assembles and outputs multiple isoforms of a particular gene (Grabherr et al. 2011). Trinity clusters the assembled transcripts into genes and their respective isoforms, which often exhibit high sequence similarity. These redundant isoforms, though biologically interesting, complicate many downstream analyses. Thus, we also generated a set of non-redundant unigenes from our transcripts using the program CD-HIT (version 4.7; Li and Godzick 2006; Fu et al. 2012) which clusters sequences by similarity using a global sequence identity threshold of 90% and keeps only the longest sequence in a given cluster. The full transcriptome assembly is available on NCBI (accession to be included pending acceptance). We also assembled each library individually, and generated library-specific sets of non-redundant unigenes with CD-HIT.

We calculated a number of statistics to determine the quality of transcriptome assembly (Table 1). We used rnaQUAST (version 4.5; Gurevich et al. 2013) to generate summary information about our assemblies, including number of contigs/scaffolds, total assembly size, maximum transcript size, median contig length, and GC content. We also ran BUSCO (version 3; Waterhouse et al. 2017), which assesses the completeness of an assembly by searching for orthologous gene sets. Using BUSCO, we determined the number of conserved orthologs from Eukaryota, Arthropoda, and Insecta that could be identified in our transcriptome.

*Genome annotation*

We used Trinotate (v. 3.0.2; Bryant et al. 2017) to annotate the transcriptome. The Trinotate pipeline integrates homology information from sequence similarity searches against known transcripts/proteins, as well as structural information from a suite of programs designed to identify specific sequence features within transcripts. We first used Transdecoder (version 3.0.1; Haas and Papanicolaou 2015) to predict likely coding regions by identifying the longest open reading frames within the set of non-redundant unigenes. Next, we ran BLASTX (Altschul et al. 1990) to align the complete unigene sequences, as well as the Transdecoder-predicted coding regions, against the UniProt/Swiss-Prot database (The UniProt Consortium 2019). Finally, we used RNAMMER (version 1.2 ; Lagesen et al. 2007) to identify rRNA transcripts, tmHMM (version 2.0; Krogh et al. 2001) to predict transmembrane regions, and SignalP (version 4.1; Nielsen et al. 1997) to locate signal peptide cleavage sites. We quantified the distribution of genes within different gene ontology (GO) categories using WEGO (version 2.0; Ye et al. 2018; Figure 2). We also assigned GO ID numbers to top-level GO categories, and plotted the results in R (version 3.3.2; R Core Team 2016) using ggplot2 (Wickham 2016).

*Gene family analysis*

We used the program OrthoFinder (version 2.3.3; Emms and Kelly 2019) to identify orthologous gene clusters among Lepidopteran transcriptomes. We used the Trinotate-derived protein set for *B. fusca*, along with proteomes for *M. sexta*, *Bombyx mori*, and *P. xylostella.* Briefly, we downloaded the assembled transcript sequences for *M. sexta* from the NCBI Transcriptome Shotgun Assembly Sequence Database (ncbi.nlm.nih.gov/genbank/tsa; Accession: GETI00000000.1) on December 2017. The transcriptome was generated from various tissue types sampled from larva, pupa, and adult individuals (Cao and Jiang 2015). For *P. xylostella*, we downloaded assembled transcript sequences from InsectBase (Yin et al. 2016; insect-genome.com) in December 2017. The transcriptomes were assembled using tissues from egg, larva, and adult individuals (He et al. 2012). For *B. mori*, we could not locate publicly available transcriptome assemblies generated from tissue samples comparable to our dataset, therefore downloaded short read data from the NCBI Short Read Archive (ncbi.nlm.nih.gov/sra; Accession: SRS241974; December 2017). These data were generated using tissue samples from embryo, larva, pupa, and adult individuals (Shao et al. 2012). We then assembled the raw *B. mori* reads using Trinity. Next, we generated sets of non-redundant unigenes for *M. sexta*, *Bombyx mori*, and *P. xylostella* transcriptomes using CD-HIT, and then identified coding regions and generated protein predictions using the program Transdecoder. Finally, we used OrthoFinder to search for orthologous gene clusters among the Trinotate-derived *B. fusca* proteins, plus protein sets of *M. sexta*, *Bombyx mori*, and *P. xylostella*. Using the set of orthologous gene clusters containing at least one gene from each species, we identified the 20 largest gene families in *B. fusca.*

*Transcriptome differences across tissue types*

Finally, to gain a preliminary understanding of transcriptome variation among tissue types, we identified the top 25 most highly expressed transcripts for each RNA library. We mapped the RNA-Seq reads from each individual library to the pooled transcriptome assembly unigenes, using the program STAR (version 2.7; Dobin et al. 2013). We calculated RPKM values (Mortazavi et al. 2008) for each transcript. In the absence of technical replicates, we were unable to assess the statistical significance of expression differences across libraries. As an alternative, we generated unique lists of the 25 transcripts with the highest RPKM values for each library type. We did this by excluding transcripts that appeared in top 25 lists for more than one library type, until each transcript occurred only a single time across all library type lists. We also identified transcripts highly abundant across the majority of RNA-Seq libraries; to do this we generated lists of 500 transcripts with highest RPKM values for each library, then identified transcripts that occurred in at least nine out of ten library’s top 500 lists.

A comprehensive TE library generated using the *B. fusca* WGS (Hardwick et al. 2019) was used as a query against the 10 individual transcriptome assemblies and the comprehensive, pooled transcriptome using command line BLAST+ with default blastn parameters. For hits that were highly identical (>95% identity and >80 bp in length), the number of unique TE that appear in each of the individual transcriptomes, and the comprehensive transcriptome, were tallied and summed for 6 categories of TEs: DNA transposons, Helitrons, LTRs, LINEs, SINEs, and unknown elements (Supplemental Table S10).

Lastly, draft genome sequences from *Cotesia sesamiae* Kitale and *C. sesamiae* Mombasa (Supplemental Data Files S4 and S5) were used as queries against the *B. fusca* genome using command line BLAST+ to identify regions of the genome that might have been horizontally transferred (HT) between these two species. Hits that were >95% identity and >80 bp in length were retained (Supplemental Data File S6). Each sequence was also used as a query to search against the individual and comprehensive transcriptome assemblies, to see if putative horizontally-transferred regions are universal or limited in their transcription. Each hit that met the thresholds for potential HT candidates described is listed in Table S11, along with the most informative hit for characterising the sequence based on a search against nt/nr and RefSeq partitions of the GenBank database using blastn and blastx with default parameters to characterize/annotate the sequences.

**Supplemental Results**

*Transcriptome assembly*

The *B. fusca* transcriptome (BioProject PRJNA553865) contains 240,022 transcripts, with the non-redundant set of unigenes containing 185,159 sequences (Table 1). Median transcript length within the unigenes was 377 bp. Our transcriptome completeness was high, with 99.6% of conserved single-copy orthologs in the BUSCO Eukaryota database recovered (Table S2; 76.2% of the Eukaryota BUSCOs were present in complete, single copies, 19.1% were present in complete, duplicated copies, and 4.3% were present in fragmented copies). In addition, we recovered 99.2% of arthropod BUSCO genes, and 98.6% of insect BUSCO genes. The median number of isoforms per gene was one for all individual libraries, with library 5_S5_L001 (infection treatment: exposed Cs-Inland) having the highest max number of isoforms for a single transcript (transcript TRINITY_DN8760_c0_g1, with 45 isoforms reported by Trinity). Furthermore, the antenna libraries (4_S4_L001 and 3_S3_L001) had the highest percentage of genes with greater than one isoform per gene (21.1% and 19.4%, respectively), while the egg library (2_S2_L001) had the lowest (6.6%).

*Genome annotation*

We annotated the assembled *B. fusca* transcriptome using Trinotate and identified 22,707 protein-coding genes, and generated a set of 39,445 proteins overall. Figure S1 gives an overview of the GO terms associated with all annotated transcripts in the *B. fusca* transcriptome assembly after 20,118 of genes were assigned to gene ontology categories using Trinotate. Briefly, for “Biological Process”, we observed the highest numbers of genes with GO IDs related to “Cellular Process” (n=21,076) and “Metabolic Process” (n=16,676). For “Cellular Component”, we observed the highest numbers of genes assigned GO IDs related to “Cell” (n=22,530) and “Organelle” (n=17,779), and for “Molecular Function”, the highest numbers of genes were assigned GO IDs for “Binding” (n=18,481) and “Catalytic Activity” (n=13,114).

*Gene family analysis*

We used OrthoFinder to search for orthologs of *B. fusca* proteins within the protein sets of *B. mori*, *M. sexta*, and *P. xylostella.* We identified 5,009 clusters shared across all species, encompassing 34,246 total proteins (9,204 of which were from *B. fusca*). The top 20 largest gene families identified by OrthoFinder are listed in Table S3. The largest gene family identified in *B. fusca* is OG0000003, which includes 30 unique *B. fusca* genes. The majority of protein sequences in orthogroup OG0000003 show significant homology to arylphorin subunit alpha (UniProt accession P14296). We also identified several large gene families with potential roles in immune function, including OG0000042, which shows significant similarity to the atlastin (UniProt accession Q9VC57); OG0000370, which shows similarity to the alaserpin (UniProt accession P14754); and OG0000052, which shows similarity to the glucose dehydrogenase [FAD, quinone] (UniProt accession P18172).

*Transcriptome differences across tissue types*

Table S4 shows a selection of transcripts that were abundant in the majority of RNA-Seq libraries, indicating ubiquitous expression across tissue types, developmental stages, and infection treatments. The majority of transcripts on this (17 out of 30) list showed significant sequence similarity to cytochrome c oxidase genes including cytochrome c oxidase subunit 1, cytochrome c oxidase subunit 2, and cytochrome c oxidase subunit 3 (Uniprot accessions P67794, P84290, P00417). We also observed expression of transcripts heat shock 70 kDa protein cognate 4, ATP synthase subunit a, 40S ribosomal protein S8, and cytochrome b (Uniprot accessions Q9U639, Q1HRS5, Q8WQI5, Q8M0K7).

To find transcripts with potentially important tissue-specific functions, we identified the top 25 unique transcripts with the highest RPKM values within tissue types/developmental stages. Table S5 shows lists of highly expressed genes for libraries from *B. fusca* exposed to *C. sesamiae* coast and inland populations. *B. fusca* exposed to Cs-Inland show highest expression of transcript TRINITY_DN26563_c133_g4, which shows significant similarity to 15-hydroxyprostaglandin dehydrogenase (UniProt accession P15428). We also observed high levels of expression of transcripts with putative immune function, including TRINITY_DN28485_c3_g4_i1, which has high homology to the gloverin (UniProt accession P86358); and TRINITY_DN20829_c3_g1_i3, which has homology to Lebocin-4 (UniProt accession O15946). In contrast, the *B. fusca* exposed to Cs-Coast show highest expression of transcript TRINITY_DN26217_c12_g4_i1, which has significant homology to the arylphorin subunit beta (UniProt accession P14297).

Table S6 shows lists of highly expressed genes for libraries from different *B. fusca* developmental stages. For the egg stage, the transcript with highest expression was TRINITY_DN26738_c6_g2_i1, which is highly similar to the apolipophorin-3 (UniProt accession P13276). For neonates, the transcript with highest expression was TRINITY_DN18637_c5_g2_i1, which shows similarity to the elongation factor 1-alpha (UniProt accession P68104). Finally, for the larva stage, the most highly expressed transcript did not have high homology to anything in the SwissProt database. However, multiple highly expressed transcripts from this library showed sequence similarity to the antimicrobial peptide Alo-1 (UniProt accession P83651).

Table S7 shows lists of highly expressed genes for libraries from different *B. fusca* tissue types. The most highly expressed transcript for antennae is TRINITY_DN20370_c10_g1_i1, which shows significant homology to replicase polyprotein (UniProt accession Q9DSN9). To investigate this result further, we ran a BLAST search of the sequence against the non redundant (nr) nucleotide database, and found a significant hit (87% identity; 92% query cover) to Iflavirus sequence (NCBI accession MG992408; Kludkiewicz et al. 2019). The antennae also showed high expression levels of multiple transcripts with homology to general odorant-binding protein 1 (UniProt accession P31418). In contrast, the ovipositor had high expression levels of transcripts with homology to the ejaculatory bulb-specific protein 3 (UniProt accession Q9W1C9). Finally, the thorax showed the highest levels of expression for transcripts with similarity to the cytochrome b (UniProt accession P00163), calcium-transporting ATPase sarcoplasmic/endoplasmic reticulum type (UniProt accession P22700), and ATP synthase subunit a (UniProt accession P35381).

Tables S8 and S9 show sex-specific differences in transcript abundance in different *B. fusca* tissues. Table S8 shows uniquely highly abundant transcripts in female versus male antennae. The majority of the uniquely expressed transcripts in *B. fusca* females were annotated as general oderant binding proteins. For example, the most abundant transcript, TRINITY_DN28049_c9_g1_i2, and had significant similarity to general odorant-binding protein 1 (Uniprot accession Q95VP3). We also observed a number of abundant transcripts in the female library with sequence similarity to pheromone binding protein (Uniprot accession Q27388). In the male antenna library, the most abundant transcript on the uniquely expressed list (TRINITY_DN27532_c101_g1_i2) did not have significant similarity to any proteins in the SwissProt database. Four transcripts (TRINITY_DN25375_c38_g1_i2, TRINITY_DN27406_c318_g1_i4, TRINITY_DN28049_c10_g1_i1, and TRINITY_DN27406_c429_g2_i2) had significant similarity to general odorant-binding proteins, including general odorant-binding protein 1, general odorant-binding protein 2, and general odorant-binding protein 83a (Uniprot accessions Q27226, Q27288, P54193).

Table S9 shows uniquely highly abundant transcripts in female versus male thoraces. The most abundant uniquely expressed transcript in the female thorax is TRINITY_DN28371_c5_g2_i1, with sequence similarity to ejaculatory bulb-specific protein 3 (Uniprot accession Q9W1C9). In the male thorax, the most abundant uniquely expressed transcript is TRINITY_DN27532_c75_g3_i1, which was annotated as a cytochrome b gene (Uniprot accession Q8M0K7).

TEs from all six categories (listed in order of abundance: LTRs, LINEs, DNA transposons, Helitrons, unknown elements, and SINEs) were transcribed in the comprehensive transcriptome, and five out of 6 categories were represented in all 10 of the individual transcriptome assemblies (SINEs, which are not numerous in *B. fusca*, were the only category of TE not transcribed in all tissues/stages; Supplemental Table S10). Interestingly, the greatest number of TEs in all 6 categories are transcribed in male antennae (2927 out of 3158 unique TEs; 93%), much higher than the numbers of unique TEs expressed in the comprehensive genome (e.g., 534 of the 663 characterized DNA transposons in *B. fusca* are transcribed in the male antennae). The transcriptome assembly with the fewest TEs transcribed in each category was the one made from egg tissue (only 55% of TEs are expressed in this tissue type). As might be expected, the more abundant a particular category of TE is in the genome, the higher the percent of TEs belonging to that category exhibiting evidence of transcription. This may be because, for some elements (e.g., LTRs), transcription is necessary for replication. In the comprehensive transcriptome, overall 80% of the TEs identified in the *B. fusca* genome were found to be transcribed (Table S10).

Using draft sequence data generated from two strains of *Cotesia sesamiae* wasps (Kitale [Supplemental Data File S4] and Mombasa [Supplemental Datafile S5]), we were able to identify candidate sequences (>95% identical over >80 bp in length) that may have been horizontally-transferred between *B. fusca* and its parasitoid wasps (Supplemental Data File S6). Of these candidates, 14 out of 17 sequences appeared in one or more of our transcriptome assemblies (Supplemental Table S11). Five of the 17 candidates were found in all 10 individual libraries. The tissue type with the greatest number of candidate HT sequences transcribed was male antennae, with 14 of the 17 candidates being expressed. We characterized these 17 candidate sequences (by using them as queries against the nr/nt and RefSeq databases), and three of them matched viral sequences, suggesting that endogenized viruses might be especially prone to horizontal transfer among species.

**Supplemental References**

Altschul, S. F., Gish, W., Miller, W., Myers, E. W., & Lipman, D. J. (1990). Basic local alignment search tool. Journal of Molecular Biology, 215(3), 403-410.

Bryant, D. M., Johnson, K., DiTommaso, T., Tickle, T., Couger, M. B., Payzin-Dogru, D., Lee, T. J., Leigh, N. D., Kuo, T. H., Davis, F. G., Bateman, J., Bryant, S., Guzikowski, A. R., Tsai, S. L., Coyne, S., Ye, W. W., Freeman, R. M. Jr, Peshkin, L., Tabin, C. J., Regev, A., Haas, B. J., & Whited, J. L. (2017). A tissue-mapped axolotl de novo transcriptome enables identification of limb regeneration factors. Cell Reports, 18(3), 762-776.

Cao, X., & Jiang, H. (2015). Integrated modeling of protein-coding genes in the *Manduca* *sexta* genome using RNA-Seq data from the biochemical model insect. Insect Biochemistry and Molecular Biology, 62, 2-10.

Dobin, A., Davis, C. A., Schlesinger, F., Drenkow, J., Zaleski, C., Jha, S., Batut, P., Chaisson, M., Gingeras, T. R. (2013). STAR: ultrafast universal RNA-seq aligner. Bioinformatics, 29(1), 15-21.

Emms, D. M., & Kelly, S. (2019). OrthoFinder: phylogenetic orthology inference for comparative genomics. Genome Biology, 20(1), 1-14.

Fu, L., Niu, B., Zhu, Z., Wu, S., & Li, W. (2012). CD-HIT: accelerated for clustering the next-generation sequencing data. Bioinformatics, 28(23), 3150-3152.

Grabherr, M. G., Haas, B. J., Yassour, M., Levin, J. Z., Thompson, D. A., Amit, I., Adiconis, X., Fan, L., Raychowdhury, R., Zeng, Q., Chen, Z., Mauceli, E., Hacohen, N., Gnirke, A., Rhind, N., di Palma, F., Birren, B. W., Nusbaum, C., Lindblad-Toh, K., Friedman, N., & Regev, A. (2011). Full-length transcriptome assembly from RNA-Seq data without a reference genome. Nature Biotechnology, 29(7), 644-652.

Gurevich, A., Saveliev, V., Vyahhi, N., & Tesler, G. (2013). QUAST: quality assessment tool for genome assemblies. Bioinformatics, 29(8), 1072-1075.

Haas, B., & Papanicolaou, A. (2015). TransDecoder (find coding regions within transcripts). Github, nd https://github. com/TransDecoder/TransDecoder.

He, W., You, M., Vasseur, L., Yang, G., Xie, M., Cui, K., Bai, J., Liu, C., Li, X., Xu, X., & Huang, S. (2012). Developmental and insecticide-resistant insights from the de novo assembled transcriptome of the diamondback moth, *Plutella xylostella*. Genomics, 99(3), 169-177.

Jiang, H., Lei, R., Ding, S. W., & Zhu, S. (2014). Skewer: a fast and accurate adapter trimmer for next-generation sequencing paired-end reads. BMC Bioinformatics, 15(1), 182.

Kludkiewicz, B., Kucerova, L., Konikova, T., Strnad, H., Hradilova, M., Zaloudikova, A., Sehadova, H., Konik, P., Sehnal, F., & Zurovec, M. (2019). The expansion of genes encoding soluble silk components in the greater wax moth, *Galleria mellonella*. Insect Biochemistry and Molecular Biology, 106, 28-38.

Krogh, A., Larsson, B., Von Heijne, G., & Sonnhammer, E. L. (2001). Predicting transmembrane protein topology with a hidden Markov model: application to complete genomes. Journal of Molecular Biology, 305(3), 567-580.

Lagesen, K., Hallin, P., Rødland, E. A., Stærfeldt, H. H., Rognes, T., & Ussery, D. W. (2007). RNAmmer: consistent and rapid annotation of ribosomal RNA genes. Nucleic Acids Research, 35(9), 3100-3108.

Li, W., & Godzik, A. (2006). Cd-hit: a fast program for clustering and comparing large sets of protein or nucleotide sequences. Bioinformatics, 22(13), 1658-1659.

MacManes, M. D. (2018). The Oyster River Protocol: a multi-assembler and kmer approach for de novo transcriptome assembly. PeerJ, 6, e5428.

Mortazavi, A., Williams, B. A., McCue, K., Schaeffer, L., & Wold, B. (2008). Mapping and quantifying mammalian transcriptomes by RNA-Seq. Nature Methods, 5(7), 621-628.

Onyango, F. O., & Ochieng ‘‐Odero, J. P. R. (1994). Continuous rearing of the maize stem borer *Busseola fusca* on an artificial diet. Entomologia Experimentalis et Applicata, 73(2), 139-144.

R Core Team. (2016). R: A language and environment for statistical computing. R Foundation for Statistical Computing, Vienna, Austria. http://www.R-project.org/.

Shao, W., Zhao, Q. Y., Wang, X. Y., Xu, X. Y., Tang, Q., Li, M., Li, X., & Xu, Y. Z. (2012). Alternative splicing and trans-splicing events revealed by analysis of the *Bombyx mori* transcriptome. RNA, 18(7), 1395-1407.

Song, L., & Florea, L. (2015). Rcorrector: efficient and accurate error correction for Illumina RNA-seq reads. GigaScience, 4(1), s13742-015.

The UniProt Consortium. UniProt: a worldwide hub of protein knowledge. (2019). Nucleic Acids Research. 47, D506–D515.

Waterhouse, R. M., Seppey, M., Simão, F. A., Manni, M., Ioannidis, P., Klioutchnikov, G., Kriventseva, E. V., & Zdobnov, E. M. (2018). BUSCO applications from quality assessments to gene prediction and phylogenomics. Molecular Biology and Evolution, 35(3), 543-548.

Wickham, H. (2016). ggplot2: Elegant Graphics for Data Analysis. Springer-Verlag, New York. ISBN 978-3-319-24277-4, https://ggplot2.tidyverse.org.

Ye, J., Zhang, Y., Cui, H., Liu, J., Wu, Y., Cheng, Y., Xu, H., Huang, X., Li, S., Zhou, A., & Zhang, X. (2018). WEGO 2.0: a web tool for analyzing and plotting GO annotations, 2018 update. Nucleic Acids Research, 46(W1), W71-W75.

Yin, C., Shen, G., Guo, D., Wang, S., Ma, X., Xiao, H., Lui, J., Zhang, Z., Liu, Y., Zhang, Y., Yu, K., Huang, S., & Li, F. (2016). InsectBase: a resource for insect genomes and transcriptomes. Nucleic Acids Research, 44(D1), D801-D807.

**
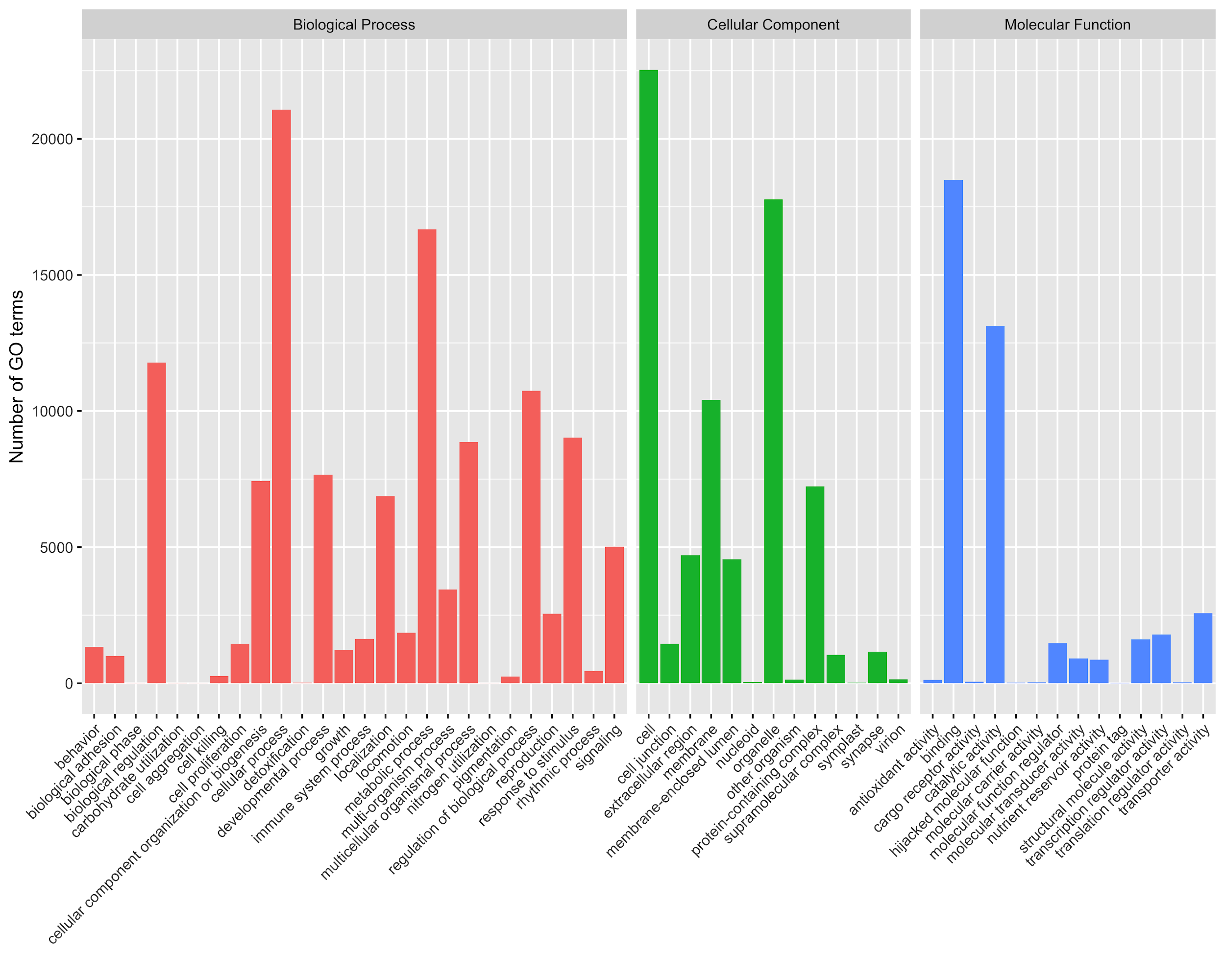
**

**Supplemental Figure 1.** GO term categories for transcripts from *Busseola fusca* annotated using Trinotate and classified into top level categories using WEGO.

**Appendix A.**  Full author list with institutional affiliations has been uploaded as a supplemental file.
