# Supplementary material for: Comprehensive Transcriptome of the Maize Stalk Borer, Busseola fusca, from Multiple Tissue Types, Developmental Stages, and Parasitoid Wasp Exposures
Source: Genome Biol Evol. 2020 Sep 18;12(12):2554–60. doi: 10.1093/gbe/evaa195 (PMC7802516; doi:10.1093/gbe/evaa195)
Supplement: evaa195_Supplementary_Data [file evaa195_supplementary_data.zip › suppl legends.docx]

**Supplemental Materials**

**Supplemental Methods**

**Supplemental Results**

**Supplemental References**

**Supplemental Figure 1.** GO term categories for transcripts annotated using Trinotate and classified into top level categories using WEGO.

**Appendix and Supplemental Tables,** uploaded separately in a single workbook:

**Appendix A.**  Full author list with institutional affiliations.

**Table S1.** Sample information for *Busseola fusca* RNA sequencing libraries.

**Table S2.** Summary statistics for transcriptomes generated from individual tissue and developmental stage-specific libraries.

**Table S3.** Twenty largest gene families in *Busseola fusca* identified by orthogroup analysis.

**Table S4.** Most abundant transcripts from across all pooled libraries prepared using *B. fusca*.

**Table S5.** Most abundant (top 25) transcripts from libraries prepared using *Busseola fusca* exposed to *Cotesia sesamiae* Mombasa and Kitale.

**Table S6.** Most abundant (top 25) transcripts from libraries prepared from different developmental stages of *Busseola fusca.*

**Table S7.** Most abundant (top 25) transcripts from libraries prepared using different tissues from *Busseola fusca*.

**Table S8.** Most abundant (top 25) transcripts from libraries prepared using *Busseola fusca* female and male antennae.

**Table S9.** Most abundant (top 25) transcripts from libraries prepared using *Busseola fusca* female and male thoraces.

**Table S10.** Transposable elements (listed by type) for which there is evidence of transcription in either the individual or comprehensive transcriptomes.

**Table S11.** Candidate horizontal transfer sequences shared between *B. fusca* and *Cotesia sesamiae* (sequenced from two strains, Kitale and Mombasa) that are transcribed in the individual and comprehensive transcriptomes.

**Supplemental Data Files,** uploaded separately in a single zipped file:

**Data File S1.** Trinity-generated file with assembled transcripts for pooled transcriptome.

**Data File S2.** Trinotate-generated file with transcriptome annotations.

**Data File S3.** Transdecoder-generated file of protein sequences.

**Data File S4.** Low coverage draft whole genome sequence of *Cotesia sesamiae* (Kitale).

**Data File S5.** Low coverage draft whole genome sequence of *Cotesia sesamiae* (Mombasa).

**Data File S6.** FASTA file of all horizontal transfer candidates between *Busseola fusca* and *Cotesia sesamiae* (Kitale and Mombasa) based on >95% sequence identity and a minimum length of 80 bp.

**All other data associated with this project can be found on NCBI under Bioproject PRJNA553865.** Sequence data and assemblies for individual libraries can be found under the following accessions: 2_S2_L001: SRR12107984, GITF00000000; 1_S1_L001: SRR12107983, GITJ00000000; S2_S1_L001: SRR12107982, GITA00000000; 5_S5_L001: SRR12107981, GITI00000000; S3_S2_L001: SRR12107980, GITB00000000; S10_S5_L001: SRR12107985, GISZ00000000; 4_S4_L001: SRR12107986, GITH00000000; 3_S3_L001: SRR12107987, GITG00000000; S7_S4_L001: SRR12107989, GITE00000000; S6_S3_L001: SRR12107988, GITD00000000. The comprehensive assembly is under accession GISL00000000.
