# Supplementary figures and images for: Comprehensive Transcriptome of the Maize Stalk Borer, Busseola fusca, from Multiple Tissue Types, Developmental Stages, and Parasitoid Wasp Exposures
Source: Genome Biol Evol. 2020 Sep 18;12(12):2554–60. doi: 10.1093/gbe/evaa195 (PMC7802516; doi:10.1093/gbe/evaa195)

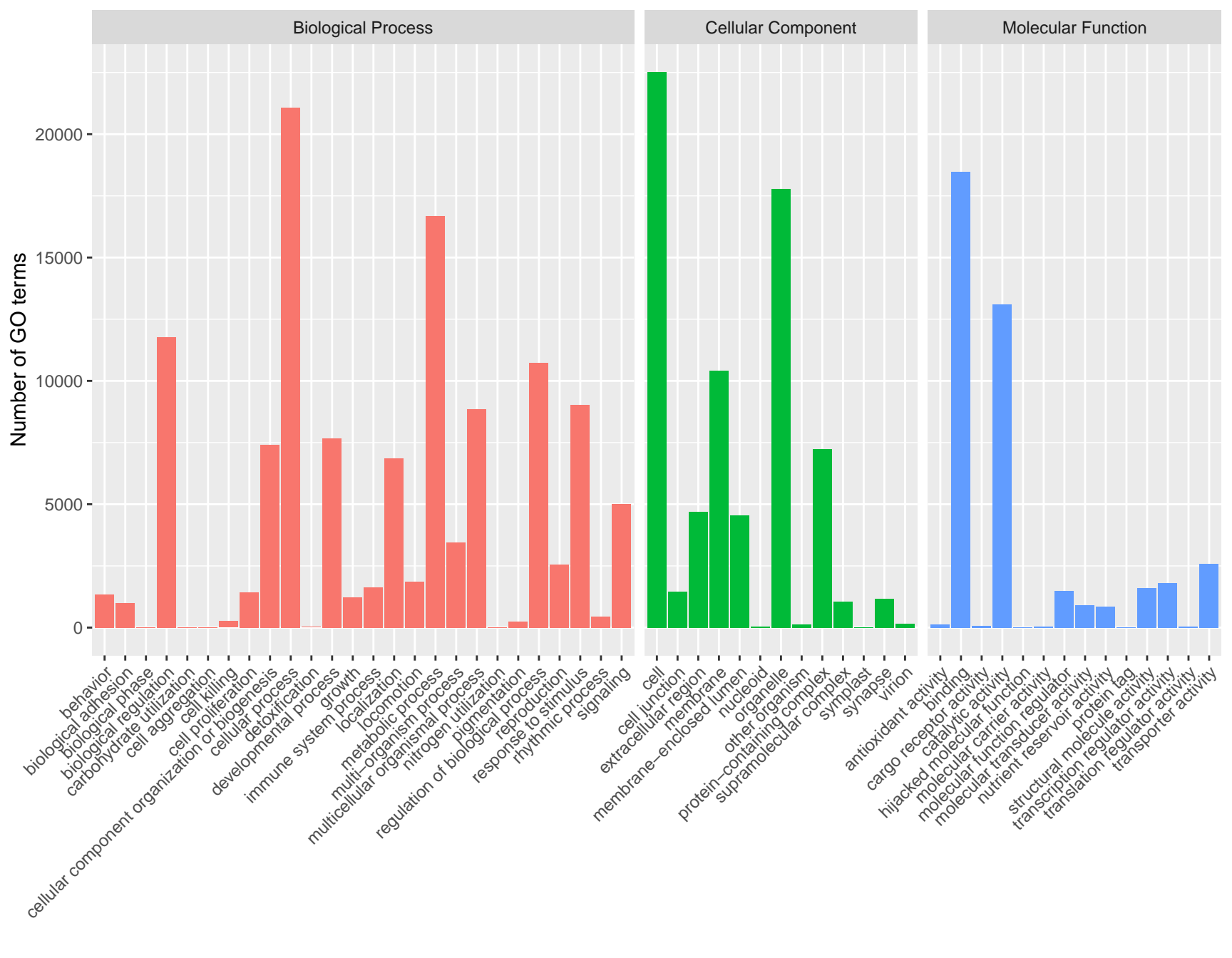

Supplement: evaa195_Supplementary_Data [file evaa195_supplementary_data.zip › FigS1_Bfusca_WEGO.pdf]
